# Characterization of the immune response elicited by the vaccinia virus L3 protein delivered as naked DNA

**DOI:** 10.1016/j.vaccine.2018.02.033

**Published:** 2018-03-07

**Authors:** Maite Ramírez, Saritza Santos, Osmarie Martínez, Ricardo Rodríguez, Eric Miranda, Willy D. Ramos-Perez, Miguel Otero

**Affiliations:** a Department of Microbiology, University of Puerto Rico, Medical Sciences Campus, San Juan, PR, USA; b University of Puerto Rico, Medical Sciences Campus, School of Medicine, San Juan, PR, USA

**Keywords:** L3L, Vaccine, Smallpox, DNA-vaccine, Novel antigen, Bioterror agents

## Abstract

Poxviruses are complex dsDNA viruses with over 200 genes, many of them with unknown role in the stimulation of immune responses. Among these, the vaccinia virus (VACV) L3L ORF encodes an essential protein for the transcription of the VACV early genes. To the best of our knowledge, the immune response elicited by L3 has not been characterized. In this regard, our data describes a DNA L3-coding plasmid (pL3L) that stimulates both, humoral- and cell-mediated immune responses in a mouse model. Cell-mediated immune responses were measured by IFN-*γ* and IL-4 ELISPOT assays. We performed CD8^+^ cells depletion and flow cytometry analysis to account for the contribution of cytotoxic T lymphocytes in the IFN-*γ* production. Moreover, results from ELISPOT were confirmed by measuring the concentration of IL-4 and IFN-*γ* in supernatant of antigen-stimulated splenocytes by cytokine ELISA. Additionally, dominant antigenic regions of L3 protein were identified by epitope mapping analysis. Humoral immune responses were assessed by ELISA. Specifically, the production of total IgG, IgG1 (TH-2) and IgG2a (TH-1) were determined one week after the final immunization. Our ELISPOT data shows pL3L-immunized animals to produce significantly higher frequencies of IFN-*γ* Spot-Forming Cells (SFC) versus controls. IL-4 levels remained unchanged in all three groups, demonstrating the increase in antigen-specific IFN-*γ* releasing cells. Flow cytometry assay results showed that CD8^+^ T cells are a major contributor to the production of IFN-*γ*. Moreover, our formulation enhances the production of total IgG, predominantly IgG2a isotype. Immunization with pL3L promotes a robust cytotoxic immune response, crucial against viral pathogens. In addition, our vaccine candidate promotes an increase in IgG levels, especially IgG2a (TH-1 type). Our data encourages further studies of L3 as a novel antigen in vaccine development against poxviruses.

## Introduction

*Poxviruses* are one of the most complex and biggest families of viruses. They consist of double stranded DNA [1] with genomes ranging from 130 to 360 kb in length [2] encoding over 200 open reading frames [3]. Their complex brick-shaped capsids are about 240–300 nm [4]. They are the only known viruses that can replicate entirely in the cytoplasm, as they possess the essential viral biosynthetic machinery for DNA and RNA synthesis [5]. Variola, Monkeypox, Cowpox, and Molluscum contagiosum viruses are among the known human pathogenic members. Infections with these agents are usually presented with a generalized rash, which is highly infectious. Poxviruses can be transmitted by zoonosis [6], contaminated fomites or objects, from person to person via air droplets [6,7], direct contact with rash [8], sexual transmission [9] and the transplacental route [10].

To prevent smallpox infection, there is only a prophylactic vaccine approved by the Federal Drug Administration (FDA). Its formulation is based on a live-vaccinia virus and thus is contraindicated for a large group of the population [11]. Serious adverse effects, including progressive vaccinia, autoinoculation, eczema vaccinatum, generalized vaccinia, congenital vaccinia, and postvaccinial encephalitis [12–14] may occur after administration to an immunologically compromised patient. For these reasons, there is an increasing need to develop safer approaches that can benefit every individual.

In the present study, we focus on the L3L open reading frame (VACWR090). L3L encodes for a 40.6 kDa protein [15], consisting of 350 amino acids and expressed in late kinetics, that is conserved in all orthopoxviruses [16]. Therefore, we expect this antigen to promote cross-protection. However, its role at eliciting an immune response remains unidentified.

## Materials and methods

### Design of the VVWR L3 DNA vaccine

The L3L gene from Vaccinia Virus Western Reserve (VVWR) used in this study was synthesized by BlueHeronBio (Bothell, WA, USA), and cloned into the pVax1 (Invitrogen, Grand Island, NY, USA) BamHI and XhoI (New England Biolabs, Ipswich, MA, USA) restriction sites, to generate the vaccine construct (pL3L). The plasmid also contains a kanamycin resistance gene, a BGH polyadenylation signal, and is under a cytomegalovirus promoter (CMV) control. Additionally, our clone has an immunoglobulin E (IgE) leader sequence, a Kozak consensus sequence, and a hemagglutinin (HA) tag (S1 File).

### Plasmid propagation and purification

Plasmids were propagated in TOP10 E. coli cells (Invitrogen, Valencia, CA, USA). Purification was assessed using the PureLink HiPure GigaPrep Kit following the manufacturer’s instructions (Life Technologies, Carlsbad, USA). Plasmids were resuspended in purified water and stored at 20 °C until the day of immunization. Characterization of purified pVAX1 and pL3L plasmids was assessed by enzymatic digestion using XhoI and BamHI (New England Biolabs, Ipswich, MA, USA), and DNA sequencing analysis (Davis Sequencing, Davis, CA, USA). Confirmation of sequence assembly was assessed using the bioinformatics software MacVector (Cary, NC, USA).

### Mice

Female 4–6-week-old BALB/c mice were acquired from Charles River (Wilmington, MA, USA). Maintenance of the animals was in accordance with the guidelines from The National Institutes of Health (Bethesda, MD, USA), and the University of Puerto Rico Institutional Care and Use Committee (IACUC). All animal tests were carried out according to the protocol [9250112], including efforts to minimize suffering of animals under study. All groups consisted of four mice, and all experiments were performed at least three times.

### Study design

Plasmids were formulated at a concentration of 1.0 µg/µL of DNA in a 150 mM sodium citrate buffer and 0.25% Bupivacaine. Animals were immunized in the quadriceps with 100 mg of the plasmids, three times by intramuscular (i.m.) injection with a 27-gauge sterile needle, at two-week intervals. Blood samples and spleens were collected one week after the last immunization.

### Synthetic peptides

The peptides used in this work were derived from the sequence of the VVWR L3 protein and synthesized as 11-mer overlapped 15-mer peptides by JPT Peptide Technologies (Berlin, Germany). These were manufactured as both: a) individual peptides for epitope mapping and b) peptide pool. L3 peptides were diluted in RPMI at a concentration of 0.5 mg/mL and stored at ‒20 °C.

### IFN-γ and IL-4 ELISPOT

Capture anti-murine IFN-γ or IL-4 antibodies (R&D Systems, Minneapolis, MN, USA) were individually coated by overnight incubation at 4 °C onto High-Protein Binding IP 96-well Multiscreen TM plates (Millipore, Bedford, MA, USA). Plates were blocked with 1% BSA for 2 h. Then, 2 × 10^5^ splenocytes per well were seeded in RPMI, and stimulated overnight (for IFN-γ) and 48 h (for IL-4) at 37 °C, in 5% CO_2_ with the VVWR L3 peptide-mix (JPT Peptide Technologies, Berlin, Germany). Concanavalin A (Con A, 5 mg/mL; Sigma-Aldrich, St. Louis MO, USA) and RPMI were used as a positive and negative controls, respectively. After stimulation, plates were washed and incubated overnight at 4 °C with biotinylated anti-mouse IFN-γ or IL-4 antibodies. Spot development was assessed after the additon of 5-Bromo-4-Chloro-3’ Indolylphosphate p-Toluidine Salt (BCIP) and Nitro Blue Tetrazololium Chloride (NBT) (R&D Systems, Minneapolis, MN). An automated ELISPOT reader system (CTL analyzers, Cleveland OH, USA) was used for spot quantification using the ImmunoSpot software (CTL analyzers, Cleveland OH, USA). The mean number of spots from triplicate wells was adjusted to 1×10^6^ splenocytes. Antigenspecific responses to IFN-γ and IL-4 were obtained after deducting the number of spots formed in the wells containing the RPMI alone from the spots developed in response to the L3 peptides.

### CD8^+^ cell depletion

Pools of splenocytes from individual groups were incubated with anti-mouse-CD8^+^ antibodies and separated as recommended by the manufacturer’s instructions using the autoMACS magnetic cell separation system (Miltenyi Biotec, Bergisch Gladbach, Germany). Briefly, mouse spleen cells were incubated with CD8a (Ly-2) MicroBeads (Miltenyi Biotec, Bergisch Gladbach, Germany). After incubation, cells were washed with 2 mL of buffer (0.25% BSA, 0.5 M EDTA), and centrifuged at 300 × *g* for 10 min. Pellets were resuspended in buffer and samples were loaded in the autoMACS.

### Flow cytometry analysis

Flow cytometry analysis was performed before and after cell removal. Cells were labeled with an antibody cocktail containing: murine anti-CD3-FITC (clone 145–2C11) from BD Biosciences (San Jose, CA, USA) and anti-CD8-PerCP/Cy5.5 (clone 53–6.7) from BioLegend (San Diego, CA, USA). Briefly, pools of splenocytes from each individual group were plated in a 96-round bottom plate at a concentration of 1 × 10^6^ cells/mL. Cells were harvested by centrifugation at 887 x g for 1 min at 4 °C. Afterwards, pellets were resuspended in 100µL FACS staining buffer (0.5 mM EDTA, 0.05% FBS, 1X PBS) containing the antibody cocktail. Labeling was performed at 4 °C, for 20 min in dark. After incubation, FACS staining buffer was added and cells were harvested under the same conditions. Examination was assessed with BD FACSCalibur cell analyzer (BD Biosciences, San Jose, CA, USA). Results were analyzed using FlowJo software (Ashland, Oregon, USA).

### Cytokine concentration in cell supernatant

Results from IFN-γ and IL-4 ELISPOT assays were validated by evaluating the cytokine profile in the cell culture supernatant. For this purpose, we used the Quantikine Mouse IFN-γ, and IL-4 sandwich immunoassays (R&D Systems, Minneapolis, USA) following a previously established protocol [17]. Optical density was measured at 450 nm in a microplate reader. Sample values were correlated to a standard curve.

### L3 immunodominant epitopes

Individual 15-mer peptides from VVWR L3L antigens were used as specific stimulators in an ELISPOT assay designed to identify the immunodominant regions. Fragments that produced a positive signal were identified by the appearance of spots in its ELISPOT well. The stimulating peptides inducing a significant increase in the frequency of Spot Forming Cells (SFC) per million splenocytes from immunized mice compared to controls were considered dominant. These *ex vivo* results were confirmed with the *in silico* identification of the dominant regions, using the computer algorithm from the BioInformatics and Molecular Analysis Section (BIMAS) of the Center for Information Technology from the NIH [18].

### Total IgG and antibody isotyping ELISA

Humoral immune responses were assessed by indirect EnzymeLinked Immunosorbent Assay (ELISA). Total IgG, and antibody isotypes were measured from mouse serum. Briefly, 50 µg of L3 antigenic protein was dissolved in 50 mM carbonate buffer to coat Maxisorp (Millipore, Bedford, MA, USA) plates. After overnight incubation at 4 °C, plates were washed. To avoid unspecific interactions, wells were blocked with ChonBlock Buffer (Chondrex, Redmond, WA, USA) for two hours at room temperature. Serum samples were diluted at 1:50 in ChonBlock and incubated for two additional hours at room temperature. Plates were washed after incubation, and HRP-conjugated goat anti-murine IgG, or IgG1, or IgG2a antibodies (Jackson Immunoresearch, West Grove, PA, USA) were diluted in ChonBlock detection antibody dilution buffer (Chondrex, Redmond, WA, USA). Samples were incubated at room temperature for sixty minutes in secondary antibody diluted at 1:2,500. Chemiluminescence produced by the secondary antibody was measured at 450 nm using a plate reader after addition of OPD peroxidase substrate (Sigma Aldrich, St. Louis, MO, USA). The reaction was stopped using 50 µL of 3 M HCl.

### Compliance with ethical standards

All protocols for mice experiments were reviewed and approved by the University of Puerto Rico Institutional Care and Use Committee (IACUC) (9250112). All the tests were carried out in strict accordance with The National Institute of Health guidelines for animal care.

### Statistical analysis

Experimental data was obtained for at least three replicates of each immunization study. The results are presented as the average ± standard error of the mean (SEM) using GraphPad Prism, version 7.02 (San Diego, CA, USA) for all analyses. The statistical significance of the tests was determined using the Kruskal-Wallis nonparametric test followed by the Dunn’s multiple comparisons test when the null model was rejected. For the depletion assay, the nonparametric Wilcoxon matched-pairs signed rank test was applied. A *p* value of 0.05 was considered significant. For all figures, *p* values are noted as *p* < 0.05 (*), *p* < 0.01 (**), *p* < 0.001 (***) or *p* < 0.0001 (****).

## Results

### Restriction enzyme digestion of pL3L

Enzymatic digestion using XhoI and BamHI and agarose electrophoresis separation showed the expected 1.1 Kbp band (Fig. S1). Moreover, sequencing analysis and assembly with a bioinformatics software confirmed the sequence.

### VVWR L3-specific IFN-γ and IL-4 responses

**Fig. 1.**
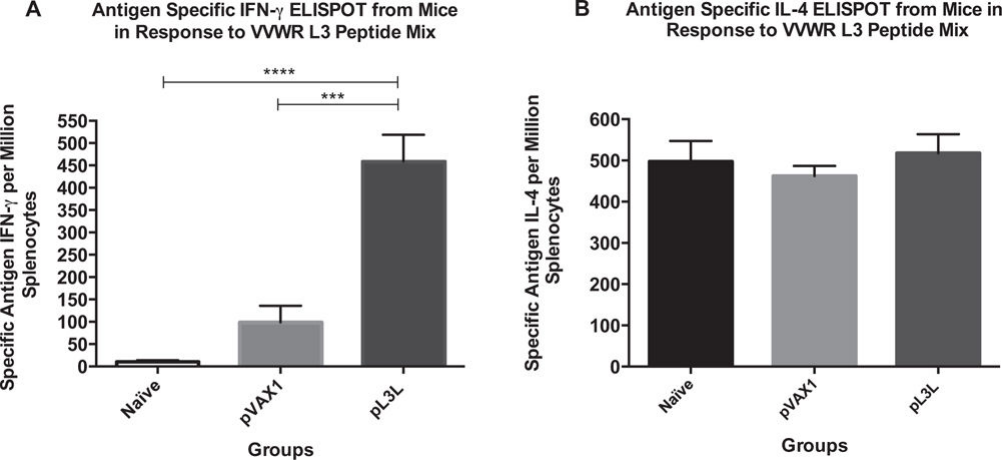
VACV L3-specific cell-mediated immune response determined by ELISPOT analysis. Each group consisted of four 4–6-week-old female Balb/c mice immunized three times, two weeks apart via intramuscular (i.m.) injection as follows: Naïve (negative control), pVax1 (backbone control), and pL3L. (A) Antigen-specific IFN–*γ* response to VVWR L3 peptides after overnight incubation. (B) Antigen-specific IL-4 response to VVWR L3 peptides after 48 h of incubation. Data are averages of at least three independent experiments, and the immune responses among groups of mice are presented as the mean ± standard error of the mean (SEM). A *p* value of <0.05 was considered significant. Con A data not shown as its values are out of scale.

The capacity of L3 to induce a cell-mediated immune response was assessed by measuring the antigen-specific release of IFN-*γ* and IL-4 from mice splenocytes. Pools of splenocytes from each immunized group were stimulated with an overlapping-peptide mix that represents the whole sequence of the VACVWR L3 protein. Frequency of L3-specific IFN-*γ* or IL-4 producing cells was determined by ELISPOT. Our data shows that immunization with pL3L induces an extremely significant increase in the production of IFN-*γ* when compared to controls. Mean frequencies of SpotForming Cells (SFC) per million splenocytes were 459.1 ± 59.6, 98.9 ± 37.0 and 9.6 ± 4.1 (ANOVA test, KWT = 29.77, p < 0.0001), for mice immunized with pL3L, pVax1, and naïve, respectively (Fig. 1A). In the case of IL-4 stimulation induced by our vaccine formulation, pL3L, pVAX1 and naïve immunized groups showed mean frequencies of 517.8 ± 45.83, 462.2 ± 24.5, 497.2 ± 50.0 SFC per million splenocytes, respectively (Fig. 1B). There was no statistical difference among them (ANOVA test, KWT = 1.045, p = 0.5930). These data demonstrate that administration of L3 as naked DNA promotes antigen-specific IFN-γ secretion.

### Contribution of CD8^+^ T cells in IFN-γ production

**Fig. 2.**
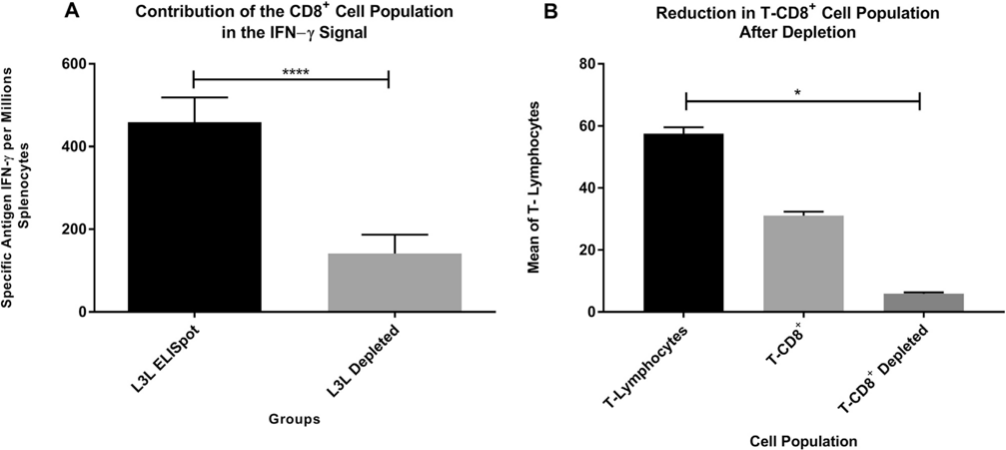
Contribution of CD8^+^ T cells in IFN-*γ* production. (A) Contribution of CD8^+^ Cell Population to IFN-*γ* production: Pools of splenocytes were incubated with anti-mouseCD8^+^ antibodies and magnetically separated using the autoMACS. Magnetically retained CD8a^+^ cells are eluted as the positively selected cell fraction. (B) Flow cytometry analysis: an 83.9% decrease in the CD8^+^ T cells population was observed after magnetic separation and results were analyzed by FlowJo. Each experiment was conducted independently at least three times and the immune responses among groups are presented as the mean ± standard error of the mean (SEM). A *p* value of <0.05 was considered significant. Con A data not shown as its values are out of scale.

Our CD8^+^ T-cell depletion assay revealed a significant decrease in the frequency of IFN-γ producing cells after CD8^+^ cell removal, compared to the non-depleted fraction (Fig. 2A). The mean frequency of Spot-Forming Cells (SFC) per million splenocytes was 84.74 ± 25.9 for the CD8^+^-depleted groups versus 459.1 ± 59.6 from non-depleted samples (Fig. 2A) (Wilcoxon matched-pairs signed rank test, W = 157, p < 0.0001).

Flow cytometry analysis was performed in both samples to determine the percentage of CD8^+^ cells before and after separation. Our results showed that 57.5 ± 2.03 percent of the cells isolated from mice spleen were T-lymphocytes (CD3^+^), from which 31.07 ± 1.27% were CD8^+^ T cells. After depletion, there was an 83.9% decrease of the CD8^+^ T cell population (ANOVA test, KWT = 7.26, p = 0.0211) (Fig. 2B). This data demonstrates a correlation between IFN-γ production and CD8^+^ lymphocytes, suggesting they play a pivotal role in the cytokine production.

### Cytokine concentration in cell supernatant

**Fig. 3.**
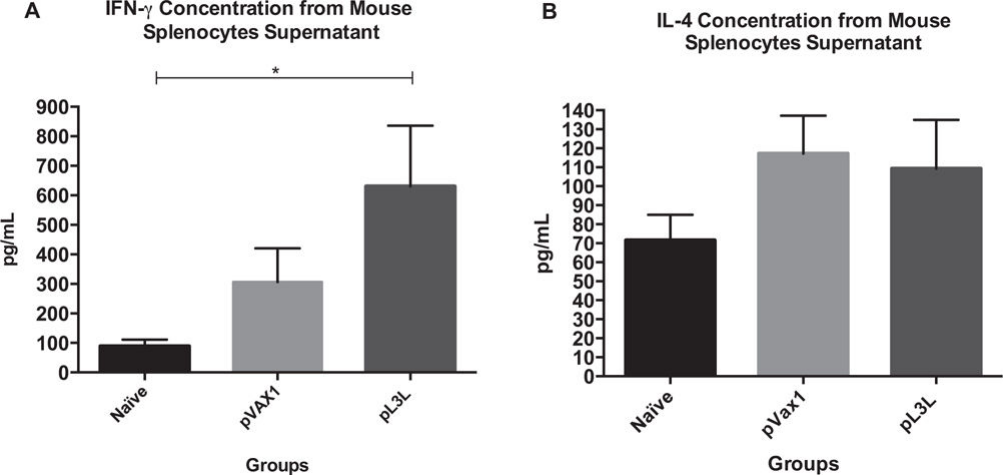
L3L-specific cytokine profile. Mice were immunized three times via i.m injection, two weeks apart from each dose. Splenocytes isolated from immunized mice were stimulated with VVWR L3 peptide pool for 5 days. Supernatants were collected, and cytokine ELISA was performed. Our data shows a significant increase in the production of IFN-*γ* in mice receiving pL3L when compared to non-immunized groups (p = 0.0215). Meanwhile, the production of IL-4 was not altered and all groups had similar values. The immune responses among groups are presented as the mean ± standard error of the mean (SEM) of at least three independent experiments. A *p* value of <0.05 was considered significant.

IFN-*γ* and IL-4 ELISPOT results were confirmed by measuring the concentration of these cytokines in supernatant of cultured mice splenocytes by ELISA. Our data shows that mice immunized with pL3L produced 630.4 ± 205.7 pg/ml of IFN-*γ* versus 306.4 ± 113.9, and 89.14 ± 22.33 pg/ml in pVAX1 and naïve groups, respectively (ANOVA test, KWT = 7.678, *p* = 0.0215) (Fig. 3A).

Furthermore, IL-4 levels were similar in all groups, showing no statistical significance among them (ANOVA test, KWT = 2.489, p = 0.3393) (Fig. 3B). These results correlate with the ones obtained from our ELISPOT, demonstrating that our formulation promotes a robust IFN-γ response.

### Activation of the humoral immune response

**Fig. 4.**
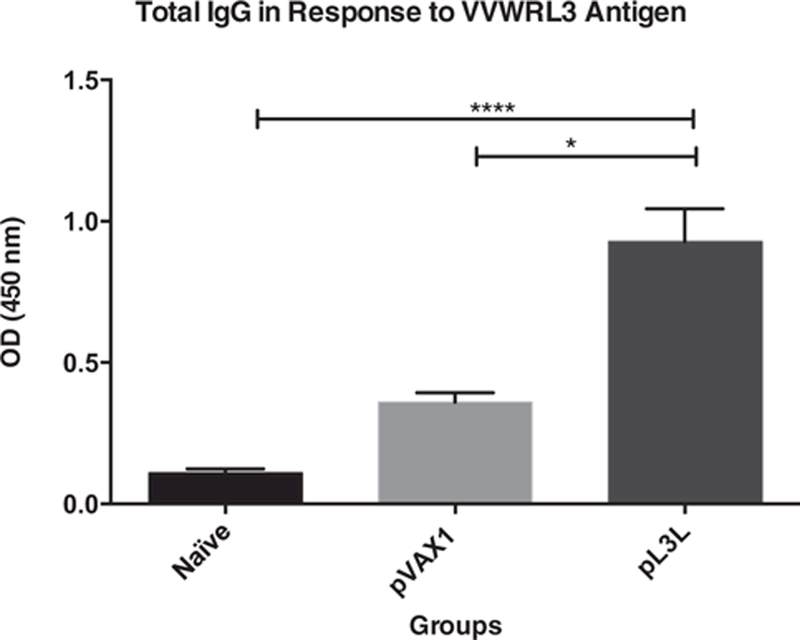
Total IgG Response Against L3 VVWR Antigen. Serum was isolated from mice one week after the final immunization. Total IgG responses were assessed by ELISA. Our data shows a significant increase in the production of total IgG in animals receiving pL3L when compared to both controls (ANOVA test, KWT = 33.31, p < 0.0001). Data is presented as the mean ± standard error of the mean (SEM) of at least three independent experiments.

To further examine the immunogenicity of this novel poxviral protein, we analyzed serum samples by indirect ELISA. The purpose of these experiments was to determine if immunization with a plasmid coding for L3 can activate antibody immunity in our animal model (Fig. 4).

Our results show that the production of total IgG (0.9274 ± 0.1163) increased 8.4- and a 2.6-times in animals that received pL3L, compared to naïve (0.111 ± 0.01274) and backbone vector (0.3599 ± 0.03356) control groups, respectively (ANOVA test, KWT = 33.31, p < 0.0001).

### Antibody isotyping

**Fig. 5.**
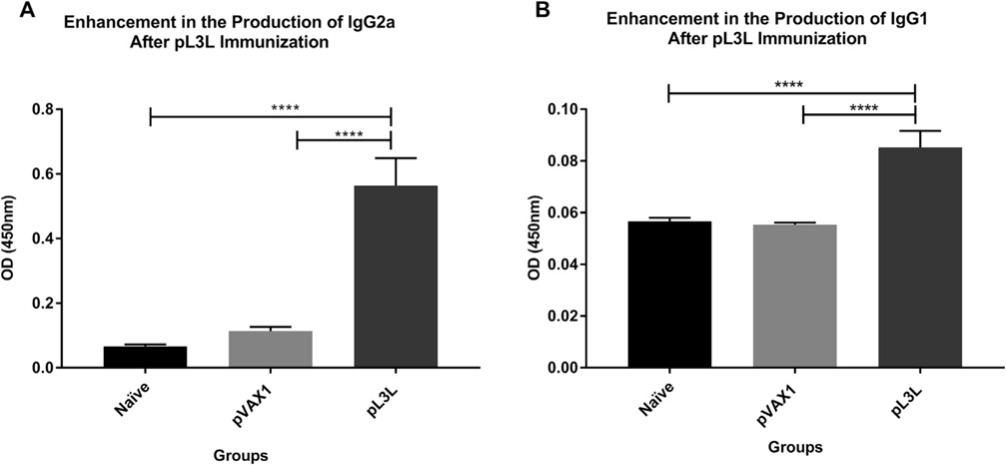
Enhancement in the production of IgG isotypes. Serum was isolated from mice one week after the final immunization. IgG isotyping was assessed by ELISA using a secondary (A) murine anti-IgG2a or (B) murine anti-IgG1 antibodies. Our data demonstrates an enhancement of both isotypes, especially IgG2a (ANOVA test, KWT = 20.64, p< 0.0001). Immune responses among groups are presented as the mean ± standard error of the mean (SEM) of at least three independent experiments.

Next, we wanted to determine which IgG isotype was pre-dominant. For this purpose, we used a secondary murine anti- IgG1 or IgG2a antibodies, with the same parameters employed to assess total IgG. Our results show that IgG2a (Th-1 type associated antibody) was significantly higher in pL3L immunized animals, 0.5632 ± 0.0856 versus 0.0661 ± 0.0061 and 0.1135 ± 0.0130 for naïve and pVAX1 groups, respectively (ANOVA test, KWT = 20.64, p < 0.0001) (Fig. 5A).

Meanwhile, IgG1 levels in pL3L-immunized animals were also higher than control groups. Means corresponding to naïve, pVAX1 and pL3L were as follows: 0.0567 ± 0.001324, 0.0553 ± 0.0008 and 0.0852 ± 0.00635 (ANOVA test, KWT = 17.09, p = 0.002) (Fig. 5B). These results demonstrate that our formulation up-regulates the production of both soluble isoforms. Moreover, the levels of IgG2a in sera were 6.6-times higher compared to IgG1, demonstrating the predominance of this isotype.

### L3 immunodominant epitopes

**Fig. 6.**
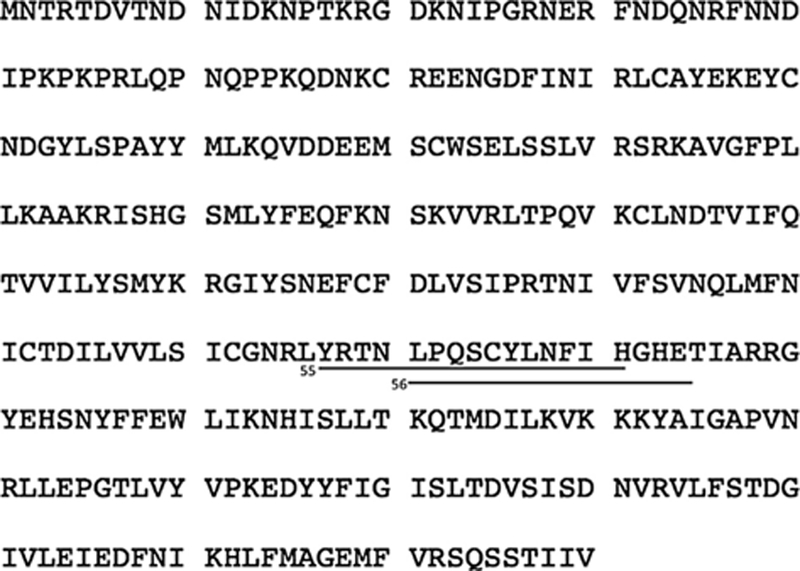
Protein localization of VACV L3 immunodominant peptides identified by ELISPOT analysis. Antigen-specific IFN–*γ* response to VVWR L3 individual 11-mer overlapped 15-mer -peptides was measured after overnight incubation. Data are averages of at least three independent experiments, and the immune responses among mouse groups are presented as the mean ± standard error of the mean (SEM). A *p* value of <0.05 was considered significant.

Once we demonstrated that a plasmid coding for the VACVWR L3 protein induced a cell-mediated immune response using a mixture of overlapping peptides, we decided to identify immunodominant regions, using the same immunization scheme. We stimulated pools of splenocytes isolated from immunized mice with individual overlapping peptides corresponding to L3 protein. The frequencies of SFC per million splenocytes producing IFN-γ were measured by ELISPOT analysis. From the 85 peptides that constituted the VVWR L3 protein sequence (Fig. 6), we observed a mean frequency of Spot-Forming Cells (SFC) per million splenocytes of 257.5 ± 99.1 and 321.7 ± 122.6, in splenocytes stimulated with peptides 55 (YRTNLPQSCYLNFIH) and 56 (LPQSCYLNFIH- GHET), respectively. Stimulation with these peptides showed a significant increase (ANOVA test, KWT = 237.3, p < 0.0001) in the frequency of Spot-Forming Cells (SFC) per million splenocytes in samples from immunized animals (55*p* = 0.0137 and 56*p* = 0.0013) when compared to the other L3 peptides. Moreover, *insilico* analysis using a computer algorithm showed the region NLPQSCYL to produce the higher score (24.000), among the L3 protein. With this information, we describe a dominant epitope region of the L3 protein in our mouse model.

## Discussion

Zoonotic poxviruses represent a serious health threat, and the number of reported cases has been increasing in recent years [6,19]. Even though ACAM2000 has demonstrated to be an extremely effective vaccine [20,21], the formulation can cause serious health complications, especially in immunocompromised individuals. Currently, around 10 million individuals in the USA are immunocompromised, and live attenuated vaccines are contraindicated for them [22]. In the eventuality of exposure to any poxvirus, this group is highly vulnerable to infection, further jeopardizing their well-being. For these reasons, it is very important to create a new formulation that can be suitable for everyone. The presented work was directed to study the immune potential of a novel poxviral protein. However, additional studies are required in order to determine the efficacy in protection of this prototype vaccine.

We tested the ability of our formulation to induce antigen- specific cytokine production. IFN-γ is the cytokine responsible for activating naïve T cells into TH-1 effectors [23,24], which is crucial to fight intracellular pathogens. Meanwhile, IL-4 polarizes the response towards a TH-2 type [25]. In this regard, our IFN-γ ELISPOT demonstrates that pL3L dramatically stimulates antigen- specific IFN-γ producing cells. The scenario was different when assessing the IL-4 response, as no variation among the three groups was observed. These results are similar to the ones obtained from the cytokine ELISA

We used depletion experiments to demonstrate that the L3L- specific IFN-γ response is mediated through CD3^+^/CD8^+^ lymphocytes. Flow cytometry analysis indicated that we obtained 83.9% of depletion, leading to an 81.5% reduction in peptide-induced IFN-γ (Fig. 3B). It has been well recognized that CD8^+^ T-lymphocytes are crucial for immune control of viral replication [26]and HIV viral load decrease [27]. This is also true during Ectromelia virus infection, as mice lacking CD8^+^ T cells have a 100% mortality rate [28]. Additionally, CD8^+^ T cells-deficient vaccinia virus-infected mice present severe cachexia, pulmonary inflammation, viral dissemination, and 100% mortality [29]. This information establishes the important role of the CTL subset in the recovery from poxviral infections. Our results show that when the amount of CD8^+^ T cells present in the ELISPOT plate decrease to less than 20%, there is a corresponding drop in the Spot Forming Cells per million Splenocytes producing IFN-γ.

Furthermore, we determined the efficacy of pL3L in stimulating antibody production. IgG is the most abundant immunoglobulin in serum and has the capacity to neutralize viral particles, activate complement, and promote phagocytosis by opsonization [30]. Our ELISA shows that mice receiving pL3L have a significant increase in total IgG production. Additionally, IgG isotype analysis shows that both, IgG2a and IgG1 levels are increased after pL3L administration.

Lastly, we performed dominant L3-epitope mapping with individual, overlapping peptides encompassing the entire L3 protein. Our analysis revealed that the region NLPQSCYL, is contained among two out of eighty-five individual peptides promoting significant release of IFN-*γ*. This information presents the first step for developing an epitope-based vaccine containing the L3 T-cell dominant epitopes.

Our study demonstrates that this novel poxviral protein can induce both humoral- and cell-mediated immune responses in mice. Our data suggests that L3 could be a promising antigen for vaccine development against poxviral infections.

## Supplementary Material

1

2
