# Supplementary figures and images for: Characterization of the immune response elicited by the vaccinia virus L3 protein delivered as naked DNA
Source: Vaccine. Author manuscript; Available in PMC 2018 Jul 30. (PMC6065253; doi:10.1016/j.vaccine.2018.02.033)

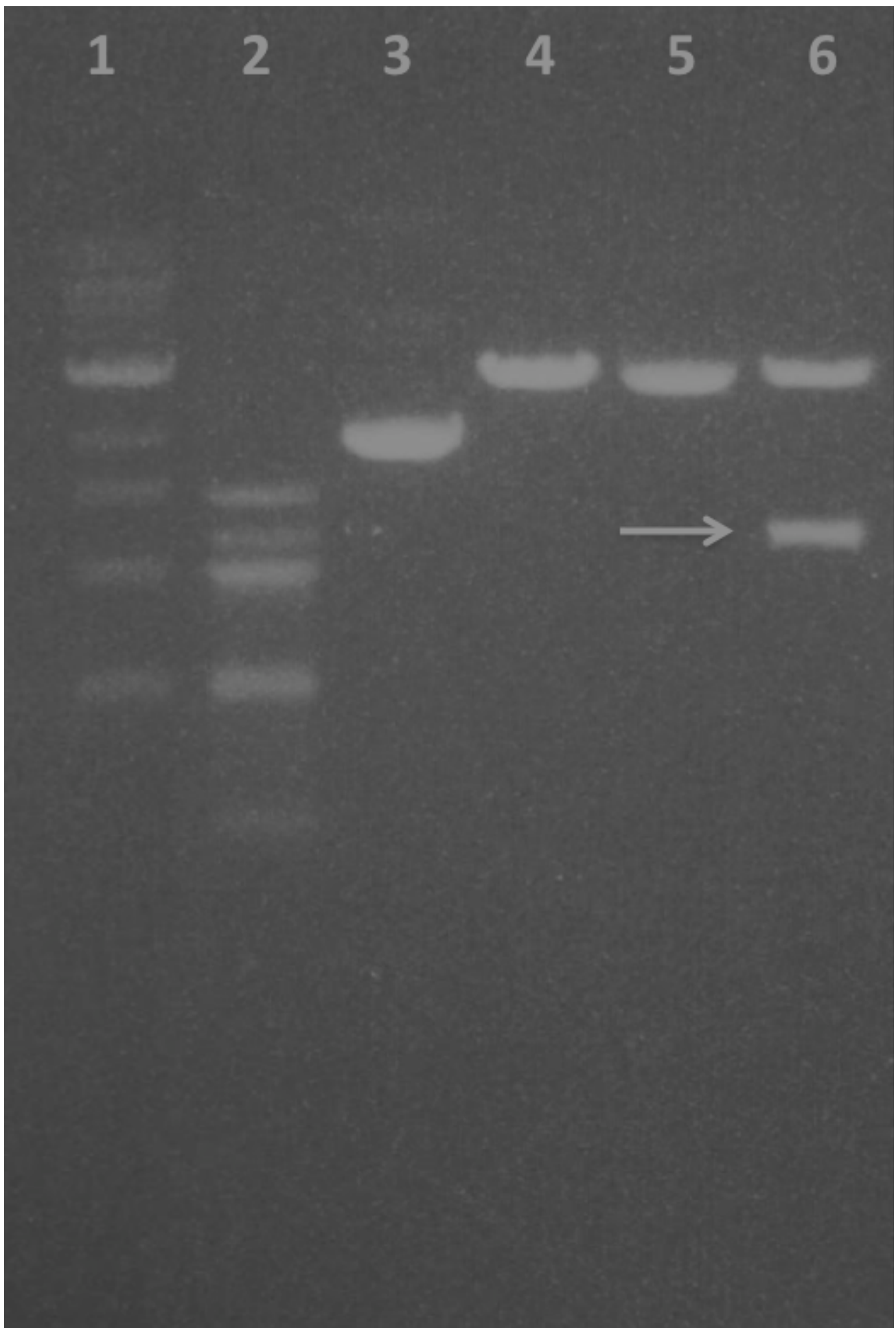

Supplement: 1 [file NIHMS955610-supplement-1.pdf]
