# Supplementary material for: Characterization of the immune response elicited by the vaccinia virus L3 protein delivered as naked DNA
Source: Vaccine. Author manuscript; Available in PMC 2018 Jul 30. (PMC6065253; doi:10.1016/j.vaccine.2018.02.033)
Supplement: 2 [file NIHMS955610-supplement-2.docx]

**S1 File. VVWR L3L gene sequence**

Yellow: BamH1restriction site; Blue: Kozak sequence; Red: IgE Signal Peptide; Green: HA tag; Purple: Xho1 restriction site; Pink: L3L STOP codon

**GGATCCGCCGCCACCATGGATTGGACTTGGATCTTATTTTTAGTTGCTGCTGCTACTAGAGTTCATTCT**AATACCCGTACCGATGTTACAAACGATAATATAGACAAAAATCCAACCAAACGAGGTGATAAAAATATACCAGGAAGAAATGAAAGATTTAATGACCAAAATAGATTCAACAACGATATACCAAAGCCTAAACCAAGACTACAGCCTAATCAGCCACCGAAACAAGATAATAAATGCAGAGAAGAGAATGGAGATTTTATCAATATTAGATTGTGTGCCTACGAGAAGGAATATTGCAATGACGGATATCTATCTCCTGCCTATTATATGTTAAAACAGGTGGATGATGAAGAAATGAGTTGCTGGTCAGAACTATCGTCGTTGGTGAGATCCAGAAAGGCGGTGGGATTTCCTCTATTAAAGGCGGCTAAACGTATTTCTCACGGATCTATGCTATATTTTGAACAGTTCAAAAACAGTAAAGTTGTGAGATTAACCCCGCAAGTTAAATGTTTAAATGATACTGTTATTTTTCAAACTGTAGTTATTTTATATTCAATGTATAAACGTGGCATATATTCTAACGAATTTTGTTTTGATCTGGTTTCTATTCCCAGAACGAACATTGTTTTTTCTGTTAATCAATTAATGTTTAACATTTGTACAGACATATTGGTAGTTCTATCTATTTGCGGCAACCGGCTCTATAGAACAAATCTACCACAGTCGTGTTACTTAAATTTCATACACGGCCATGAGACAATAGCCCGTAGAGGATATGAACACTCCAATTACTTTTTCGAGTGGTTGATAAAAAATCACATATCGCTATTGACCAAGCAAACGATGGATATTCTCAAGGTAAAGAAAAAGTATGCTATAGGAGCACCAGTAAATAGGTTGTTAGAACCTGGTACACTGGTATATGTGCCCAAAGAAGATTATTACTTTATAGGCATATCACTCACCGATGTGTCAATTAGCGATAATGTCAGAGTATTATTTTCCACAGATGGAATAGTGTTAGAAATAGAAGACTTTAATATCAAGCATTTATTTATGGCAGGTGAGATGTTTGTTAGAAGTCAGTCTAGTACTATTATAGTA**TACCCATACGACGTCCCAGACTACGCTTAACTCGAG**
